# Clinicopathological features of corneal invasion by filtering bleb

**DOI:** 10.1186/s12886-023-02859-8

**Published:** 2023-03-27

**Authors:** Minghua Shi, Hezheng Zhou, Simin Chen, Zuohong Wu, Zhong Sun, Ying Zhang, Wanju Yang, Yiqiao Xing

**Affiliations:** Aier Eye Hospital of Wuhan University, No. 481, Zhongshan Road, Wuhan City, 430000 Wuchang District China

**Keywords:** Filtering bleb, Filtering surgery, Complication, Glaucoma, Cornea, Acupuncture separation

## Abstract

**Purpose:**

To describe the clinicopathological characteristics and explore the possible etiology of cornea invasion by filtering bleb (CIFB) after filtering surgery.

**Methods:**

We reviewed 22 patients treated for CIFB between March 2005 and March 2022. The patients were followed up for more than 1 year. Slit-lamp examination, optical coherence tomography (OCT), ultrasound biomicroscopy, and histopathological examination were performed to observe the morphology of the bleb and depth of corneal invasion. Depending on the severity of the lesion, treatments consisting of local massage, acupuncture separation, or surgical resection were administered.

**Results:**

The mean age of the patients was 56.3 ± 8.8 years. All patients underwent filtering surgery in the moderate or advanced stage of glaucoma. The filtering bleb was closely connected with the cornea, and its posterior boundary was locally adhered. Forward displacement of the internal opening of the filtering bleb was found in 4 of 7 surgically treated patients. OCT and pathological examination showed that the filtering blebs invaded the corneal stroma. Removal of the adhesion of the posterior boundary of the filtering bleb by different treatment methods successfully improved the patients’ conditions.

**Conclusion:**

Filtering blebs can invade the corneal stroma. Adhesion of the posterior boundary and forward displacement of the internal opening of the filtering bleb are the possible causes of CIFB. Removal of the adhesion of the posterior boundary of the filtering bleb can halt the progression of CIFB.

**Supplementary Information:**

The online version contains supplementary material available at 10.1186/s12886-023-02859-8.

Filtering surgery is the main surgical method used to treat glaucoma. However, this surgery is associated with numerous bleb-related complications, including excessive bleb function, bleb leakage, bleb infection, and bleb scarring [1, 2]. The incidence of bleb migration onto the cornea (BMOC) is relatively low, and thus far, no accurate reports of its incidence rate are available. In patients with BMOC, the filtering bleb crosses the corneoscleral junction and enters the corneal region, causing a plugging sensation, tears, increased intraocular pressure (IOP), and other symptoms. In severe cases, the pupil is blocked, resulting in a decrease in the best-corrected visual acuity (BCVA) [3–5].

In patients with BMOC, the bleb usually covers the corneal surface, has a clear boundary with the corneal stroma, and is easy to separate from the cornea. Most authors call this type of bleb as an overhanging filtering bleb (OFB) [4–7]. OFBs can be easily separated from the cornea during surgical resection, without injuring the corneal stroma. Postoperatively, the healing is relatively good, and complications such as corneal scarring and corneal astigmatism are rare.

In 2009, our research team reported a case of a giant filtering bleb invading the cornea; the bleb had penetrated the corneal stroma and formed a tight connection with the cornea, and was difficult to separate from the cornea during surgery. We called this type of lesion “corneal invasion by filtering bleb” (CIFB) [8]. Some authors have reported a few cases of similar lesions, which they termed dissecting blebs [9]. Compared to OFB, CIFB is rare, with only a few cases having been reported [9, 10]. Moreover, we have observed that in the early stage of CIFB, some characteristic clinical manifestations can be used to differentiate CIFB from OFB, such as the conjunctival settlement line, limited adhesion of the posterior boundary of the bleb, a tight connection between the filtering bleb and the cornea that cannot be pushed forward, and a serrated anterior wall of the filtering bleb. We detect early-stage CIFB by using these typical manifestations. In the past 17 years, we have treated 22 patients with CIFB. Here, we report their clinical characteristics, pathological manifestations, and treatment outcomes, and discuss the possible causes of this complication in order to inform clinical decision-making.

## Materials and methods

### Patients

We reviewed the data of 22 patients who developed CIFB after undergoing filtration surgery for glaucoma in our hospital between March 2005 and March 2022. 12 of these patients had undergone the original filtering surgery in other hospitals. The selection criteria were mainly based on the typical clinical characteristics of CIFB. These characteristics have been described in detail in the Results section. The patients were followed up in our hospital for more than half a year after the development of CIFB.

We collected the systemic and ocular histories of all patients, including the following parameters: type of glaucoma, courses of treatment, type of operation, intraoperative medication, time interval between glaucoma surgery and onset of CIFB, and clinical symptoms. The eye examination included BCVA measurement, IOP measurement, slit lamp examination, and fundus examination. All patients underwent fluorescence staining of the filtering bleb, and the morphology, position, size, and activity of the bleb were observed and recorded, along with any leakage and adhesions with surrounding tissues. Ultrasound biomicroscopy (UBM) and/or optical coherence tomography (OCT) were used to examine the morphology of the filtering bleb in order to determine whether the bleb had migrated under the corneal epithelium. All patients were followed up and the development of the filtering bleb was dynamically observed. Patients with simple OFBs or blebs with no tight connection with the cornea were not included in this study. The study was performed in accordance with the Declaration of Helsinki and was approved by the ethics committee of Aier Eye Hospital of Wuhan University, China (approval no.: 2020IRBKY0306).

According to the extent of corneal invasion, the lesions were classified as follows: mild CIFB, a < 1.0 mm breadth of the corneal limbus was invaded by the filtering bleb; moderate CIFB, the breadth of limbal invasion was > 1 mm, but the bleb did not reach the pupil; and severe CIFB, invasion of the pupil area.

### Therapeutic methods

Treatment methods were selected according to the size of the invading bleb, the patient’s symptoms, and their willingness to undergo treatment. In principle, asymptomatic and mild CIFB can be treated using local massage. Severe CIFB was treated using surgery. Moderate CIFB was treated with acupuncture separation. During the treatment, it is important to release the adhesion of the posterior boundary of the filtering bleb.

Acupuncture separation: After inducing surface anesthesia, we separated the connecting tissue between the filtering bleb and sclera with a 30G needle. After releasing the adhesion of the posterior boundary of the filtering bleb, we maintained an open channel for the backward drainage of aqueous humor to prevent corneal invasion and migration of the filtering bleb towards the cornea. We, then, injected 0.2–0.4 mL of 25 g/L 5-fluorouracil (5-FU) next to the drainage channel, and massaged the filtering bleb backward and upward. This treatment was administered once a week for 3–5 weeks. The patients were followed up for more than 3 months.

Surgical treatment: Under local anesthesia, the filtering bleb tissue was sharply dissected to separate it from the cornea and then resected. Any scarring or adhesions between the filtering bleb and the scleral surface were separated and released. After careful observation and assessment of the shape, position, and filtration rate of the existing filtering channel, the filter bleb was repaired or reconstructed, and a drainage device was implanted if required. Depending on the condition of the corneal wound, a corneal bandage lens, an amniotic membrane covering, or lamellar keratoplasty was used.

### Statistical analysis

SPSS *v*23.0 statistical software was used for statistical analysis. Data on age and follow-up duration are expressed as x ± s. Differences in IOP before and after the treatment were compared using the paired-samples *t*-test. *P* < 0.05 was considered to indicate statistical significance.

## Results

### General information

Between March 2005 and March 2022, 22 patients who developed CIFB after undergoing filtering surgery for glaucoma were treated in our hospital. These patients consisted of 12 men and 10 women, with an average age of 56.3 ± 8.8 years (range, 44–73 years). The CIFB was judged as mild, moderate, and severe in 11, 7, and 4 patients, respectively. The glaucoma stage according to the visual field of the affected eye was advanced in 15 patients and moderate in 7 patients. The filtering surgeries in the affected eye consisted of trabeculectomy (*n* = 20), non-penetrating trabeculectomy (*n* = 1), and glaucoma drainage device implantation (*n* = 1). In all patients, filtering surgery was performed with a fornix-based conjunctival incision, and mitomycin C was used during the operation. The mean time interval between the filtering surgery and the onset of CIFB was 22.41 ± 23.99 months (range, 2–96 months). All patients underwent anterior segment photography, and 8 patients underwent OCT and/or UBM (Table 1).

**Table 1:** General information of the 22 study patients

Patient	Sex	Age (yrs)	Eye with CIFB	Type of glaucoma	Visual field staging of glaucoma	Anti-glaucoma surgery
1	M	58	Right	POAG	Advanced	Glaucoma drainage device implantation
2	F	64	Right	POAG	Moderate	Trabeculectomy
3	F	50	Right	PACG	Moderate	Trabeculectomy
4	F	62	Right	PACG	Advanced	Trabeculectomy
5	M	72	Right	POAG	Advanced	Trabeculectomy
6	F	49	Left	PACG	Moderate	Trabeculectomy
7	F	40	Right	POAG	Moderate	Trabeculectomy
8	F	44	Left	PACG	Absolute	Trabeculectomy
9	M	59	Left	POAG	Advanced	Trabeculectomy
10	F	58	Right	PACG	Moderate	Trabeculectomy
11	M	65	Left	PACG	Moderate	Trabeculectomy
12	M	53	Left	PACG	Advanced	Trabeculectomy
13	M	56	Right	Secondary glaucoma after ICE	Advanced	Trabeculectomy
14	F	73	Right	PACG	Advanced	Trabeculectomy + orthotopic autologous trabecular transplantation
15	F	47	Right	PACG	Advanced	Trabeculectomy
16	M	62	Left	POAG	Advanced	Trabeculectomy
17	F	54	Right	PACG	Advanced	Trabeculectomy
18	M	64	Right	POAG	Advanced	Trabeculectomy
19	M	47	Left	PACG	Moderate	Trabeculectomy
20	M	46	Left	POAG	Advanced	Non-penetrating trabecular surgery
21	F	55	Left	POAG	Advanced	Trabeculectomy
22	F	61	Left	PACG	Advanced	Trabeculectomy

*CIFB* Corneal invasion by filtering bleb, *M* Male, *F* Female, *PACG* Primary angle-closure glaucoma, *ICE* Iridocorneal endothelial syndrome, *POAG* Primary open-angle glaucoma

### Treatment results

The average follow-up duration was 29.64 ± 18.67 months (range, 10–61 months). Among the 22 patients, 7 patients with mild CIFB received only local massage for 4–6 months, and their symptoms did not progress. These patients had no obvious symptoms of ocular discomfort, so they discontinued the treatment. In the remaining 4 patients with mild CIFB, the symptoms were not completely controlled after local massage for 3–6 months. These 4 patients with progressive mild CIFB and another 3 patients with moderate CIFB were treated with 3–5 sessions of acupuncture separation combined with 25 g/L 5-FU injection. One patient with severe CIFB and absolute glaucoma refused surgical treatment, and underwent acupuncture separation. After treatment, the symptoms of these 8 patients were controlled, and the anterior margin of bleb even retreated by 0.3–0.5 mm in 2 patients with moderate CIFB.

**Fig. 1:**
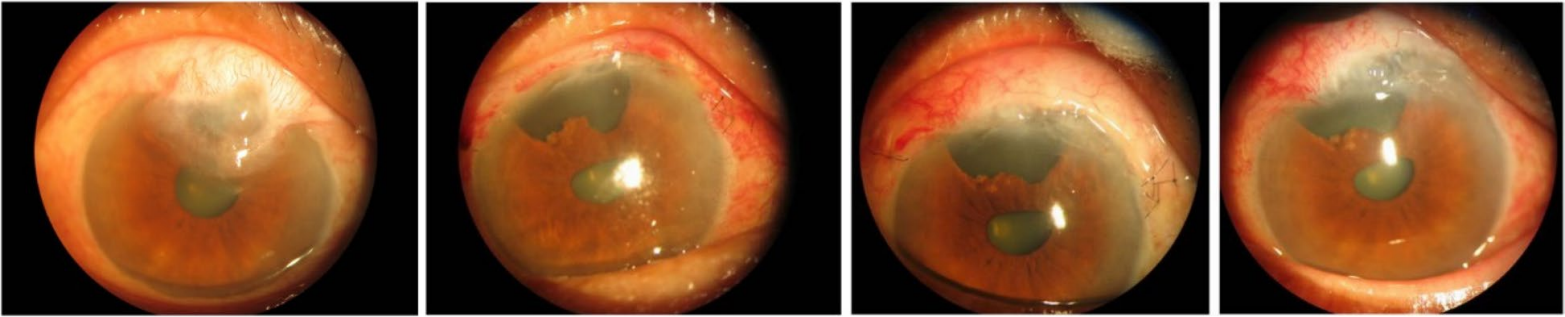
Preoperative and postoperative photos of a typical case (patient 1) From left to right are photos taken before the operation, and at 5 days, 2 months, and 1 year after the operation. The cornea is transparent, and the filtering bleb is good after the operation. However, large-scale iris defects and pupil deformation are present. At 2 months after the operation, the posterior boundary of the filtering bleb developed adhesions, and invaded into the corneal surface again. After loosening the posterior adhesion and applying local massage, the patient’s condition became stable. No significant progression has occurred during 1 year after the operation

The remaining 4 patients with moderate CIFB and 3 patients with severe CIFB underwent surgical treatment, which included excision of the corneal filtering bleb, repair or reconstruction of the filtering bleb, and implantation of a drainage device (patient #5), followed by corneal bandage lens application (2 patients), amniotic membrane transplantation (3 patients), or local lamellar keratoplasty (2 patients) to repair the corneal wound. In the 2 patients with corneal bandage lens application, the wound healed with the formation of a nebular corneal opacity. The remaining 5 patients were observed to have a transparent cornea that was free of any scar formation. After the above surgical treatments, the filtering blebs were low, flat, and diffuse in all 7 patients, with the exception of one patient (patient #5) who developed a recurrence of CIFB after 2 months. The condition of this patient stabilized after more than 2 months of frequent bleb massage (Fig. 1). No other complications were found, such as corneal infection, filtering bleb adhesion, and increased IOP. The treatment methods and patient outcomes are shown in Table 2.

**Table 2:** Time, degree, treatment method, and outcome of CIFB

Patient	Time^a^(mo)	CIFB stage	Supplementary examinations	Treatment method	Treatment results	Follow-up (mo)
1	36	Progressive, moderate	Anterior segment photography,UBM,OCT	B + C	Healing, corneal opacity	24
2	12	Progressive, mild	Anterior segment photography	S + M	Stable	60
3	14	Progressive, mild	Anterior segment photography	S + M	Stable	14
4	8	Moderate	Anterior segment photography	S + M	Stable, filtering bleb regressed	20
5	24	Severe	Anterior segment photography, UBM, OCT, surgical video	B + A + F + E	Relapse, stable after treatment	24
6	10	Progressive, mild	Anterior segment photography	S + M	Stable	30
7	14	Moderate	Anterior segment photography	S + M	Stable	16
8	36	Severe	Anterior segment photography, UBM	S + M	Stable	23
9	11	Progressive, mild	Anterior segment photography	S + M	Stable	36
10	12	Mild	Anterior segment photography	M	Stable	48
11	2	Progressive, moderate	Anterior segment photography,UBM,OCT	B + C	Healing, corneal opacity	48
12	6	Moderate	Anterior segment photography	S + M	Stable, filtering bleb regressed	24
13	96	Progressive, moderate	Anterior segment photography, UBM	B + C	Healing, corneal transparency	10
14	72	Severe	Anterior segment photography,UBM,OCT,surgical video	B + L + F	Cured, corneal graft transparent	61
15	9	Mild	Anterior segment photography	M	Stable	24
16	13	Mild	Anterior segment photography	M	Stable	20
17	12	Mild	Anterior segment photography	M	Stable	46
18	10	Mild	Anterior segment photography	M	Stable	18
19	60	Severe	Anterior segment photography,UBM,OCT	B + L + F	Cured, corneal graft transparent	36
20	14	Progressive, moderate	Anterior segment photography,UBM,OCT	B + A + F	Healing, corneal transparency	14
21	15	Mild	Anterior segment photography	M	Stable	20
22	7	Mild	Anterior segment photography	M	Stable	36

*CIFB* Corneal invasion by filtering bleb, *B* Bleb excision, *L* Lamellar keratoplasty, *F* Filter reconstruction, *A* Amniotic membrane covering, *S* Acupuncture separation, *M* Local massage, *C* Corneal bandage lens, *E* Ex-PRESS P200 glaucoma shunt implantation

^a^Time interval from glaucoma surgery to the onset of CIFB

### BCVA and IOP before and after treatment

The standard logarithmic visual acuity chart was used to record the BCVA before and after treatment. At the time of the last follow-up examination, the BCVA remained unchanged in 8 patients, was improved in 12 patients (by 1 line in 9 patients and by 5 lines in 3 patients with severe CIFB, from 0.2 to 0.6, from 0.08 to 0.4, and from manual to 0.3), and decreased slightly in 2 patients (by 1 line in 1 patient and by 2 lines in the other patient).

The mean IOP before and after treatment was 14.68 ± 3.33 and 14.09 ± 2.51 mm Hg, respectively. There was no significant difference in IOP before and after treatment (*t* = 1.930, *P* > 0.05).

### Typical appearance of CIFB on slit lamp examination

**Fig. 2:**
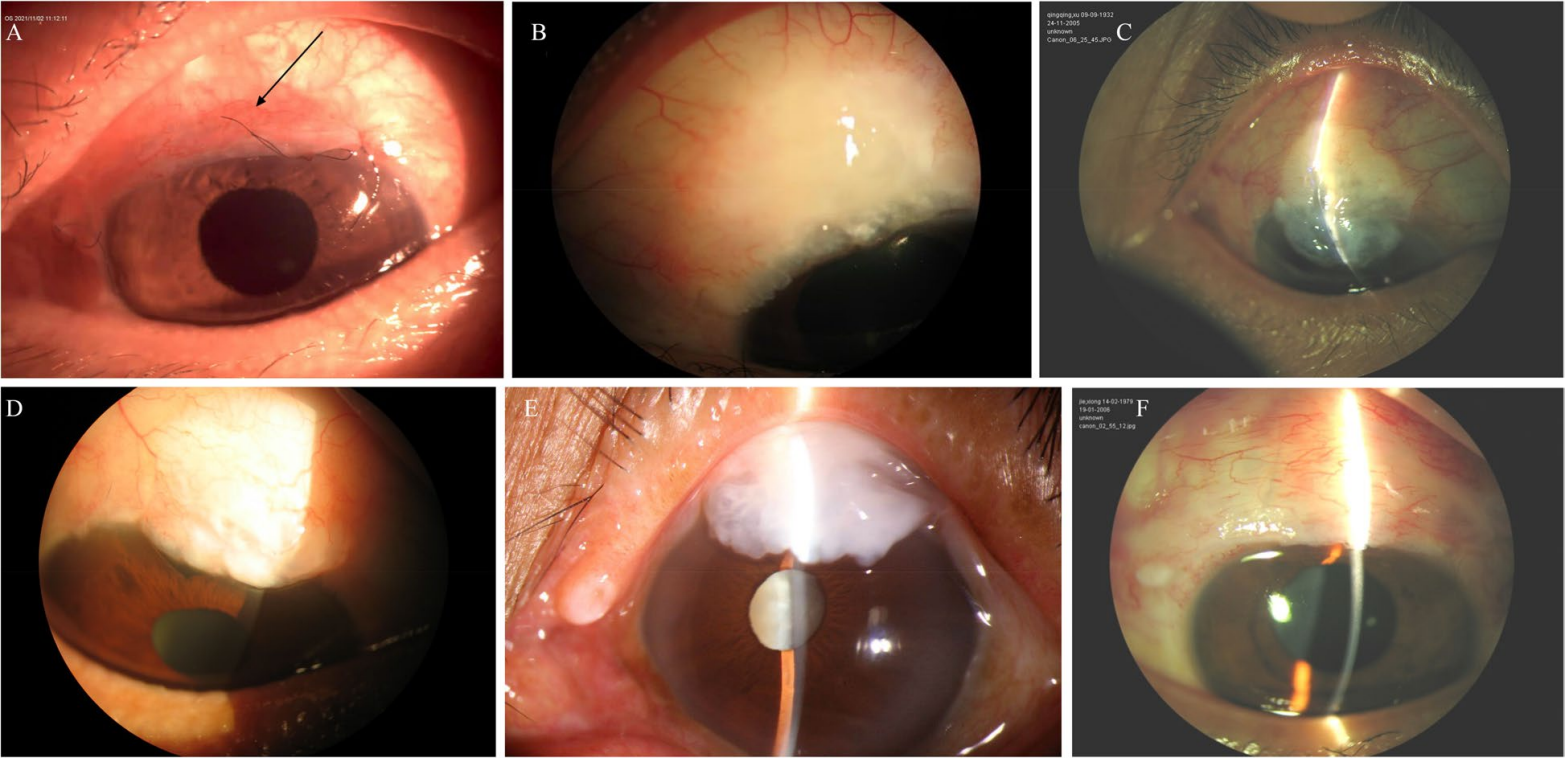
Appearance of corneal invasion by filtering bleb (CIFB), overhanging filtering bleb (OFB), and giant filtering bleb on slit lamp examination. **A**: Early-stage CIFB. In the early stage after filtering surgery, the subconjunctival tissue at the posterior boundary of the filtering bleb exhibits an adhesion, leading to the sinking of the conjunctiva and the formation of a conjunctival settlement line (arrow). **B**: Moderate CIFB. The posterior boundary of the bleb is obviously limited due to adhesion, and the anterior boundary is serrated and invades into the corneal stroma. The filtering bleb has no sagging feeling and cannot be pushed. **C**: Severe CIFB. The bleb is tightly attached to the cornea. There is no sagging feeling, and the bleb cannot be pushed. **D**: Moderate OFB. The bleb hanging over the corneal surface can be pushed upward, and the light slit is bent in a barbed shape at its lower edge. **E**: Severe OFB. The bleb is partially fused with the corneal epithelium, and serrated adhesion can be seen at its anterior boundary. **F**: Giant filtering bleb. The bleb has expanded over a large area without any obvious adhesion. The anterior boundary has crossed the limbus and entered the corneal area, and adhered with the corneal surface to some extent

Before the occurrence of CIFB, a conjunctival subsidence line was observed at the posterior boundary of the filtering bleb in some patients. We speculate that the reason for its formation was that the conjunctiva at the posterior boundary of the filtering bleb was adhered to the sclera; the local conjunctiva was sunken in an arc or groove shape, and a bent light band could be seen under the slit lamp (Fig. 2A). In patients with moderate CIFB, the filtering bleb crossed the corneoscleral junction and invaded the cornea in the form of multiple serrations. The filtering bleb on the corneal surface was flat and had no pocket-like space. It could not be pushed, and the light band was not bent at its lower edge (Fig. 2B). In patients with severe CIFB, the filtering bleb could cover the pupil area and appeared polycystic, but still did not have a pocket-like shape. Blood vessels could be seen on the surface of the bleb (Fig. 2C).

### Typical appearance of CIFB on UBM and OCT

**Fig. 3:**
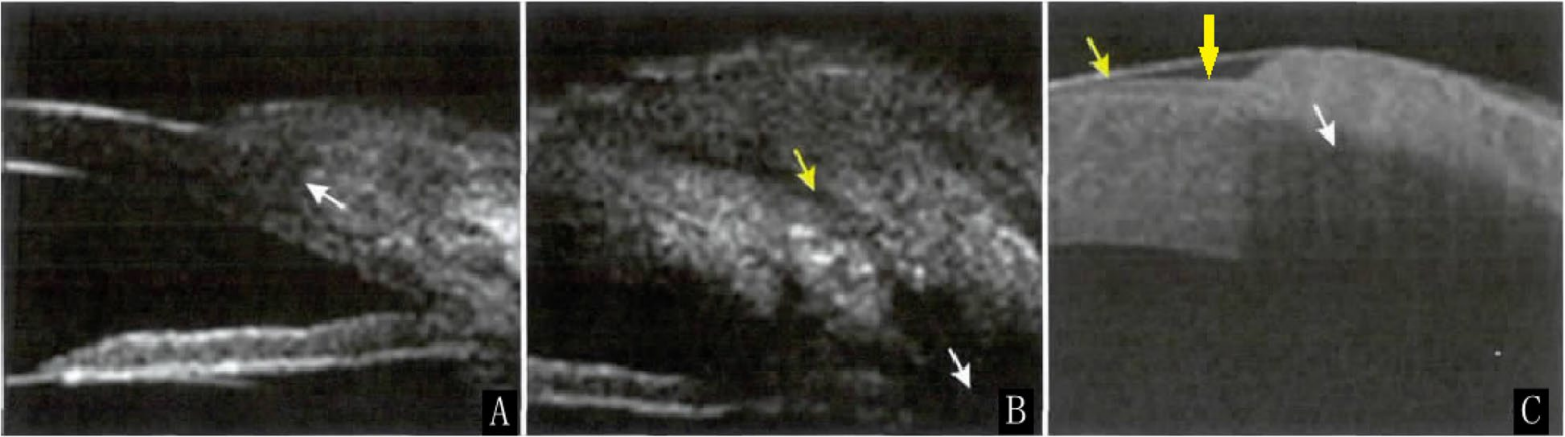
Ultrasound biomicroscopy (UBM) and optical coherence tomography (OCT) examination of a filtering bleb invading the cornea. **A**: UBM shows that the tissue on the corneal surface is a cystic structure, and the inner wall is hyperechoic. The corneal epithelial structure has disappeared, and the boundary between the inner wall of the filtering bleb and the cornea is unclear (white arrow). **B**: The echo intensity in the filtering bleb varies, with a few liquid dark areas (yellow arrow). The filtering bleb at the corneal limbus is partially unobstructed, with a liquid hypoecho in it, and the incision in the root of the iris can be seen (white arrow). **C**: OCT shows that the light reflection band of the corneal epithelium not covered by the filtering bleb is clear (yellow arrows). The corneal epithelial structure in the area covered by the filtering bleb has disappeared, and the cornea has fused with the filtering bleb (white arrow)

Of the 22 patients, 8 underwent OCT and UBM. The common manifestations of CIFB on OCT and UBM included a polycystic filtering bleb, disappearance of a component of the corneal epithelium and the Bowman membrane, and thinning of the corneal stroma in the area covered by the filtering bleb. The UBM and OCT appearance of the CIFB in patient #5 are shown in Fig. 3.

### Intraoperative appearance of CIFB

Intraoperatively, we noted that the junction between the filtering bleb and the cornea was tight. Limited adhesion was found at the posterior boundary of the filtering bleb. The filtering bleb was scarred, and it was difficult to introduce a needle into it to induce local anesthesia. It was difficult to separate the filtering blebs on the corneal surface from the cornea. In 4 of the 7 patients who underwent surgical treatment, we found that the internal opening of the filtering bleb had shifted toward the cornea. In 2 of these patients, the inner opening extended into the transparent corneal area. We repaired or reconstructed the filtering bleb to correct the obvious displacement of its internal orifice. We were concerned that the intraocular pressure after surgery was not ideal in one patient (patient #5) because of the severe surgical trauma, so we implanted an Ex-PRESS P200 glaucoma shunt for this patient. A video of the operation on patient 5 is provided in Supplementary file 1. After bleb resection, we noted that the corneal wound was thin, so we applied an amniotic membrane covering or performed lamellar keratoplasty according to the situation. The video of the operation on patient #14 is provided in Supplementary file 2.

### Histopathological features of CIFB

**Fig. 4:**
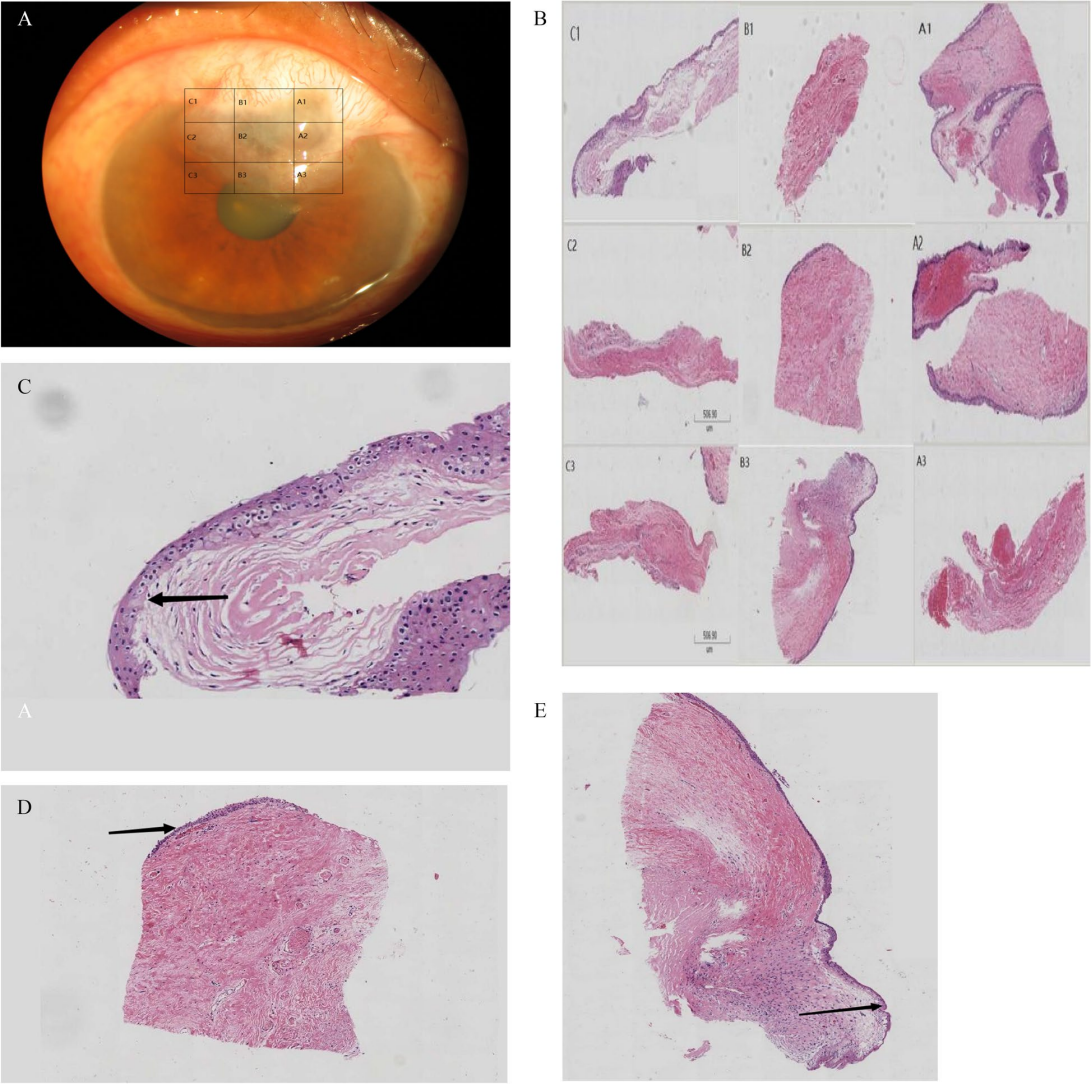
Pathological findings in a typical case (patient 1). **A** and **B**: Schematic diagrams of bleb sections and pathological examination of each section. **C**: Section C1 is located in the corneal conjunctival margin. The Bowman membrane can be seen (black arrow). **D** Section B2 is located within the corneal limbus. No epithelial tissue is present inside the filtering bleb, except for the conjunctival epithelium on the surface (arrow). The Bowman membrane has disappeared, indicating that the filtering bleb has invaded into the corneal stroma. **E** Section B3. At the lower end of the filtering bleb (at the junction with the normal corneal tissue), the corneal epithelium and conjunctival epithelium are continuous rather than in a face-to-face arrangement, indicating that the filtering bleb has invaded the cornea rather than hanging over the cornea

The surface of the resected bleb was covered with squamous epithelium, which was thickened in some areas. Proliferative capillaries, loose connective tissue, and scattered fibroblasts were sporadically distributed under the epithelium. The typical pathological examination results of patient 1 are shown in Fig. 4. The resected bleb was divided into 9 pieces. Pathological examination showed that the filtering bleb had a polycystic structure. The thickness of the cornea under the filtering bleb was 1/5^th^–3/5^th^ of that of the adjacent normal cornea. In the transparent corneal area, the Bowman membrane could be found. At the corneoscleral junction, the conjunctival epithelium and corneal epithelium were in direct continuity, and the Bowman membrane was damaged. At the part of the cornea covered by the filtering bleb, the Bowman membrane had disappeared. This indicated that the filtering bleb had invaded into the corneal stroma.

## Discussion

In this study, we have reported 22 patients who developed CIFB after undergoing filtering surgery for glaucoma, including 11, 7, and 4 patients with mild, moderate, and severe CIFB, respectively. We observed that patients with CIFB tend to have some characteristic manifestations in the early stage, such as a conjunctival subsidence line, localized adhesion of the posterior boundary of the filtering bleb, serrated invasion of the cornea, and tight adhesion of the filtering bleb with the cornea that crosses the corner of the conjunctival margin. In patients with moderate or severe CIFB, UBM, OCT, and pathological sections showed that the filtering blebs had invaded the corneal stroma. In patients who underwent surgical removal of the blebs, we found that the filter was displaced forward. To our knowledge, this is the first report to describe the clinical features of CIFB.

Anterior filtering surgery can lead to many types of bleb-related complications, including bleb dysesthesia. Bleb dysesthesia causes varying degrees of eye discomfort, but the filtration function is unaffected. This complication is associated with nasal filtering bleb, giant filtering bleb, BMOC, etc. [3, 11, 12]. OFB and CIFB are both associated with bleb dysesthesia. While nasal filtering bleb and giant filtering bleb have been extensively studied, our understanding of BMOC remains insufficient [3, 11]. Almost all cases of BMOC reported thus far have involved OFBs or giant filtering blebs. OFB refers to the expansion of the filtering bleb, which then falls or hangs off the corneal surface, forming a pocket-like space. The conjunctival epithelium of the filtering bleb adheres to the corneal epithelium. In the case of large OFBs, the corneal epithelium and conjunctival epithelium can gradually fuse due to their long-term adhesion, but the bleb does not break through the Bowman membrane and enter the corneal stroma (Fig. 2D and F) [4, 5, 8, 13, 14]. Most OFBs have a certain degree of mobility, and can be pushed easily. Their etiology is explained as follows: (1) The use of antiproliferative drugs during filtering surgery results in the thinning of the wall of the filtering bleb [5, 14–16]. (2) This thinning leads to excessive filtration or even leakage from the bleb [6, 15, 16], which then sags under the influence of gravity [15, 16] and the squeezing pressure of eyelid closure [16]. Several authors have reported that OFBs can be easily separated from the cornea during operation; blunt dissection is sufficient to accomplish this separation without causing any damage to the corneal stroma [4, 5, 13, 16, 17]. Some authors [7] have found using ultrasound and pathological examinations that the Bowman membrane under the OFB remains intact, and no channel connected with the cavity of the conjunctival filtering bleb is present, which obviates the need for suturing after the resection of the bleb. Supplementary file 3 is a surgical video of a patient with an OFB.

Earlier, it was believed that [18] because the normal corneal epithelium has a barrier effect on the conjunctival epithelium, it would be difficult for the filtering bleb tissue to invade into the cornea. In 2008, Michael DOC [9]. reported a case of a dissecting bleb. The histopathological findings suggested a subepithelial dissection component of the bleb in the cornea. In 2010, Prata et al. performed OCT for patients with OFBs, and also found that the filtering bleb tissue could invade the underlying cornea and overlying Bowman membrane [19] Al-Beshri et al. reported the immunohistochemical characteristics of 4 patients with overhanging dissecting blebs. The epithelium of the dissecting blebs showed a corneal epithelial phenotype. The immunophenotype of the extracellular matrix was similar to that of the normal conjunctival stroma, suggesting that the dissecting blebs had migrated under the corneal epithelium [10]. However, unlike our study, neither of the above studies found that the filtering bleb had entered the corneal stroma through the Bowman membrane. In some of our patients with severe CIFB, pathological examination showed that the Bowman membrane in the part of the cornea covered by the filtering bleb was destroyed and had disappeared, indicating that the filtering bleb had broken through the Bowman membrane and entered the corneal stroma. The difference between these results may be due to differences in the stage of CIFB development. In the early stage, the CIFB may not have destroyed the Bowman membrane. Unfortunately, our patients did not undergo immunohistochemical examination, and the tissue source of the bleb could not be determined.

Almost all of the patients with CIFB in this study had advanced glaucoma. It takes a long time for the filtering bleb to invade the cornea; in our study, the interval between the filtering operation and CIFB onset was 22.41 ± 23.99 months (range, 2–96 months). The appearance of CIFB under a slit lamp is obviously different from that of an OFB. Figure 2 shows a good comparison of the anterior segment photos of OFB and CIFB. In addition, among patients with severe CIFB, OCT can reveal significant thinning of the cornea that is covered by the filtering bleb and disappearance of the Bowman membrane. During surgery, the scarring of the filtering bleb can be clearly felt. It was difficult to introduce a needle into the bleb to administer local anesthetic, and blunt separation was impossible. After excision, the cornea was very thin, and an amniotic membrane covering was necessary to prevent scar formation. The postoperative pathological examination showed that the Bowman membrane of the part of the cornea covered by the filtering bleb had disappeared, indicating that the filtering bleb had invaded into the corneal stroma. As far as we know, this has not been previously reported. Supplementary files 1 and 2 show the surgical videos of patients 5 and patients 14, respectively. Supplementary file 3 is a surgical video of a patient with an OFB. With these videos, we can see the difference between CIFB and OFB more intuitively.

The mechanism of bleb invasion into the cornea is not clear. Most researchers believe that it is closely related to the surgical incision and the use of antiproliferative drugs such as mitomycin C [5, 14–16]. In our study, all patients underwent filtration surgeries with fornix-based conjunctival flaps, and mitomycin C was commonly used, which is consistent with previous reports [5, 14–16]. In addition, all our patients had advanced or moderate glaucoma. The methods of anti-glaucoma surgery for such patients are more complex, such as autologous trabecular transplantation. Even trabeculectomy is a complex surgery in such patients, as it requires a fornix-based conjunctival flap, mitomycin C infiltration, adjustment of sutures, and continuous sutures for the conjunctival incision. Thus, there is greater trauma to the local tissues, which induces local tissue hyperplasia and scar formation, resulting in adhesions and hyperplasia of the filtering bleb [9].

We also found that most of our patients had limited adhesion at the posterior boundary of the bleb. The conjunctival subsidence line at the trailing edge of the filtering bleb is a characteristic manifestation of early adhesion formation (Fig. 2F). This type of bleb needs to be distinguished from giant filtering blebs, which are usually well-functioning, but cause discomfort to the patient by interrupting the tear film distribution over the bleb and cornea. Giant filtering blebs can cross the limbus and cover the cornea [3, 11, 12]. The etiology of giant filtering blebs is similar to that of CIFB. Although giant filtering blebs occupy a wide area, the degree of invasion into the cornea is less, and the blebs do not show significant adhesion. In contrast, in CIFB, the posterior boundary of the bleb shows limited adhesion, and the anterior aqueous humor cannot diffuse to the surrounding conjunctival sac, resulting in bleb expansion in the direction of the cornea. This may be one of the main causes of CIFB. The appearance of a settlement line at the posterior boundary of the filtering bleb is a characteristic manifestation of early adhesion (Fig. 2A). The settlement line is the result of the adhesion and contraction of the local fascia and the sclera beneath it.

The therapeutic effect in some patients also supports this inference. For those with early progressive blebs, we could relieve the adhesion of the posterior boundary of the filtering bleb by using local massage and acupuncture separation, supplemented by antiproliferative drugs to prevent scar formation. This method is commonly used in patients with filtration failure caused by filtration bleb adhesion [20–22]. Here, we used this method to remove the adhesion of the posterior boundary of the filtering bleb, which successfully prevented the progression of CIFB and obviated the need for surgery. For patients with large CIFB, we performed surgical removal of the corneal filtering bleb. We carefully removed the adhesion of the posterior boundary and cut off the fibrous tissue to restore the diffusion of the filtering bleb and to prevent recurrence of CIFB.

Another phenomenon we observed in this study is that the forward displacement of the internal orifice of the filtering bleb was present in almost all patients who underwent surgery. The malposition of the internal orifice even involved the transparent cornea. The inflammatory reaction caused by continuous aqueous humor stimulation may be one of the inducements for the filtering bleb to invade the cornea. For these patients, we repaired the corneal leakage or re-established the filtering channel to eliminate the stimulation and damage caused by the aqueous humor to the corneal epithelium and to reduce the risk of recurrence of CIFB.

It is well-known that the proliferation and adhesion of filtering blebs may lead to the obstruction of the filtering port, which is an important cause of the failure of filtering surgery. In almost all of the 22 patients in this study, normal IOP was maintained after the onset of CIFB, and during the entire follow-up period. No cases of filtration failure caused by filter mouth obstruction and filter bleb leakage occurred. Previous limited case reports [3–5, 9–11, 17] have also shown that whether the filtering bleb is suspended or the filtering bleb invades the cornea, the filtration function is hardly affected. We speculate that the adhesion of the filtering bleb only occurs at the posterior boundary, and the lack of adhesion at the internal orifice allows the filtration function to continue. The displacement of the internal orifice of the filtering bleb also prevents the formation of adhesions to a certain extent, so that the filtration function is maintained. Of course, other factors may be involved, and further observation is needed.

In general, we believe that CIFB is a distinct disease entity that differs from OFB in terms of clinical manifestations, etiology, histopathology, and optimal treatment method. The filtering bleb in patients with CIFB can invade into the corneal stroma. The etiology may be related to the adhesion of the posterior boundary of the filtering bleb and the displacement of the inner orifice of the filtering bleb toward the transparent cornea. The progression of CIFB can be alleviated by removing the adhesion at the posterior boundary of the filtering bleb. The differences between CIFB and OFB have been summarized in Table 3.

**Table 3:** Comparison of corneal invasion by filtering bleb (CIFB) and overhanging filtering bleb (OFB)

	**CIFB**	**OFB**
Slit lamp examination	Conjunctival subsidence line is present in the early stage. The filtering bleb intrudes into the cornea in a zigzag shape (serrations), and is closely connected with the cornea and cannot be moved. The filtering bleb is flat without any overhang. In severe cases, the bleb can cover the pupil area, and appears polycystic without any sagging	The filtering bleb extends over the limbus, overhangs the corneal surface, forming a pocket-like structure. The sagging bleb does not break through the Bowman membrane or enter the corneal stroma. Most of these blebs can be easily moved
Ultrasound biomicroscopy and optical coherence tomography	The filtering bleb is polycystic. The corneal epithelium and Bowman membrane disappear in the area covered by the filtering bleb, and the corneal stroma becomes thinner	The corneal stroma does not become thinner. The Bowman membrane under the bleb is intact
Intraoperative findings	Tight adhesion between the filtering bleb tissue and the cornea and scarring are present. Needle insertion is difficult. The filtering bleb cannot be easily separated from the cornea. The posterior boundary of the filtering bleb is obviously adhered. The internal orifice of the filtering bleb moves forward toward the transparent cornea	Blunt separation can be achieved. The posterior boundary does not show adhesions, and the internal orifice of the filtering bleb is not displaced. The corneal wound remains transparent after healing
Pathological examination	The resected surface is covered with squamous epithelium, which is thickened in some areas. Proliferative capillaries, loose connective tissue, and scattered fibroblasts are present under the epithelium. The Bowman membrane is destroyed and has disappeared, and part of the corneal stroma is thinner	The Bowman membrane under the bleb is intact. No channel connected with the cavity of conjunctival filtering bleb is present

*CIFB* Corneal invasion by filtering bleb, *OFB* Overhanging filtering bleb

## Supplementary Information

**Additional file 1. **The surgical videos of patients 5: Bleb excision+filter reconstruction+ Ex-PRESS P200 glaucoma shunt implantation +amniotic membrane covering.

**Additional file 2. **The surgical videos of patients 14: Bleb excision+ filter reconstruction+ lamellar keratoplasty.

**Additional file 3.  **The surgical videos of patients with OFB.

## Data Availability

The data are available from the corresponding author upon reasonable request.

## Abbreviations

CIFB: Corneal invasion by filtering bleb
OCT: Optical coherence tomography
BMOC: Bleb migration onto the cornea
IOP: Intraocular pressure
BCVA: Best-corrected visual acuity
OFB: Overhanging filtering bleb
UBM: Ultrasound biomicroscopy
5-FU: 5-Fluorouracil
